# Screening Maize Germplasm for Resistance to Fall Armyworm (*Spodoptera frugiperda*) and Its Association with Genomic SNP Variation

**DOI:** 10.3390/genes17050526

**Published:** 2026-04-29

**Authors:** Constantino Francisco Lhamine, Arsênio Daniel Ndeve, Domingos Raquene Cugala, Pedro Fato, Pedro Silvestre Chauque, Rogério Marcos Chiulele, Suwilanji Nanyangwe, Mable Chebichii Kipkoech, Kolawole Peter Oladiran

**Affiliations:** 1 Department of Crop Production, Eduardo Mondlane University, Maputo P.O. Box 257, Mozambique; ndevegod@gmail.com (A.D.N.); chiulelerogerio@gmail.com (R.M.C.); suwilanjinanyangwe02@gmail.com (S.N.); mablekipkoech13@gmail.com (M.C.K.); oladirankolawole5@gmail.com (K.P.O.); 2 Centre of Excellence in Agri-Food Systems and Nutrition (CE-AFSN), Eduardo Mondlane University, Praca 25 de Junho Edificio da Reitoria 5° Andar, Maputo P.O. Box 257, Mozambique; dcugala@gmail.com; 3 Agricultural Research Institute of Mozambique (IIAM), Maputo P.O. Box 3558, Mozambique; chauquepedro@hotmail.co.uk; 4 Department of Crop Protection, Eduardo Mondlane University, Maputo P.O. Box 257, Mozambique

**Keywords:** maize, *Spodoptera frugiperda*, host plant resistance, AUDPC, SNP markers, maize breeding, Mozambique

## Abstract

**Background/Objectives:** Fall armyworm (FAW) (*Spodoptera frugiperda*) is a major constraint to maize production in Sub-Saharan Africa, including Mozambique. This study aimed to evaluate maize genotypes for resistance to FAW under greenhouse and field conditions and to assess the association between phenotypic resistance and genomic variation based on single nucleotide polymorphisms (SNPs). **Methods:** A total of 20 maize genotypes from the Agricultural Research Institute of Mozambique (IIAM) and the International Maize and Wheat Improvement Center (CIMMYT) were evaluated. FAW damage was quantified using the area under the damage progress curve (*AUDPC*). Phenotypic data were analyzed using ANOVA and mixed models, while molecular analysis was conducted using 10,603 SNP markers located on chromosomes previously associated with FAW resistance. **Results:** Significant genotypic differences were observed under greenhouse conditions (F = 1.94, *p* = 0.012) and in the field (*p* = 0.021), although environmental factors reduced variation in the field. Genotypes such as CML67, CML338, and Kenya amarelo (Acc3550) exhibited consistently lower AUDPC values across environments, indicating stable resistance. However, SNP allele proportion was not significantly associated with phenotypic resistance (r = 0.34, *p* = 0.147), and regression and ANOVA analyses confirmed the absence of a significant relationship (*p* > 0.05). **Conclusions:** FAW resistance in maize is quantitatively inherited and not explained by general genomic variation across candidate regions. Phenotypic screening remains essential, and further studies are required to identify specific loci for effective marker-assisted selection. The identified stable genotypes represent valuable resources for breeding FAW-resistant maize adapted to Mozambique.

## 1. Introduction

Maize (*Zea mays* L.) is one of the most important staple crops in Sub-Saharan Africa and plays a critical role in food security and rural livelihoods. In Mozambique, maize is widely cultivated by smallholder farmers and represents a major source of food, income, and household nutrition [1,2]. Across the region, maize contributes a significant proportion of daily caloric intake, making improvements in maize productivity essential for sustaining food security.

Maize production in the region is increasingly threatened by the fall armyworm (*Spodoptera frugiperda* J.E. Smith), an invasive lepidopteran pest native to the Americas. The pest was first reported in West Africa in 2016 and rapidly spread across most African countries within a short period [3,4]. Since its introduction, fall armyworm has caused severe damage to maize crops, leading to substantial economic losses and threatening smallholder farming systems across Sub-Saharan Africa.

Fall armyworm larvae feed primarily on maize leaves and whorl tissues, producing characteristic feeding symptoms such as window-pane lesions, irregular holes, and severe defoliation. These injuries reduce the photosynthetic capacity of the plant and can significantly affect plant growth and grain yield [1,5]. Yield losses associated with fall armyworm infestation have been estimated to range between 20% and 50% depending on the infestation levels, environmental conditions, and crop management practices [4].

Although chemical insecticides are commonly used to control fall armyworm infestations, their effectiveness is often limited in smallholder farming systems due to high costs, limited accessibility, and potential environmental and health risks [6,7]. Consequently, the development of maize varieties with genetic resistance to fall armyworm has emerged as one of the most sustainable and environmentally friendly approaches for long-term pest management [1].

Screening maize germplasm for resistance under both controlled and field conditions is an essential step for identifying resistant parental material for breeding programs. Controlled environments such as greenhouses allow for accurate evaluation of intrinsic resistance by ensuring uniform pest pressure, whereas field trials provide insight into resistance expression under natural environmental conditions [8,9].

In addition to phenotypic screening, advances in molecular genetics have introduced powerful tools for accelerating crop improvement. Molecular markers, particularly SNPs, enable the identification of genomic regions associated with resistance traits and facilitate marker-assisted selection in breeding programs [10]. Recent studies have identified several quantitative trait loci (QTLs) associated with fall armyworm resistance in maize, highlighting the potential of integrating molecular markers with conventional phenotypic screening to improve selection efficiency [11,12].

Therefore, the objective of this study was to evaluate maize genotypes from the IIAM and the CIMMYT for resistance to fall armyworm under greenhouse and field conditions and to identify genotypes with stable resistance across environments. In addition, molecular marker analysis was used to validate the presence of genetic factors associated with fall armyworm resistance in the evaluated germplasm.

## 2. Materials and Methods

### 2.1. Experimental Sites

**Figure 1:**
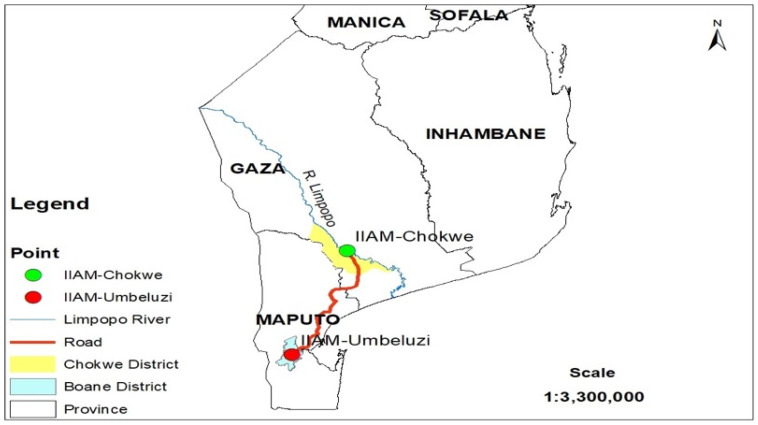
Study location.

The study was conducted at two research locations in Mozambique: Umbeluzi Research Station (Boane district, Maputo Province) and the Chókwè Agrarian Station (Chókwè district, Gaza Province) (Figure 1). Umbeluzi served as the site for seed increase of the maize genotypes used in the study. The evaluation of FAW resistance was conducted at Chókwè under both greenhouse and field conditions. Chókwè was selected because it provides suitable crop management conditions for maize experimentation, including reliable irrigation water availability, well-established experimental infrastructure, and favorable environmental conditions for maize growth and pest development. The district is located within the Limpopo River irrigation scheme, which supports irrigated crop production and allows for controlled crop management during experimental trials. In addition, the environmental conditions of the Chókwè region are suitable for maize cultivation and for the development of insect pests such as fall armyworm, enabling the effective evaluation of host plant resistance under field conditions [1,4,13].

### 2.2. Plant Material

A total of 20 maize genotypes were evaluated for resistance to fall armyworm (FAW), comprising 16 maize lines, accessions, and landraces obtained from IIAM and 4 inbred lines sourced from CIMMYT (Table 1). The IIAM maize genotypes were developed and maintained under Mozambican agroecological conditions, and selected lines have previously been used as parental material in IIAM breeding programs. These genotypes represent locally adapted germplasm with potential value for resistance breeding. The CIMMYT inbred lines, obtained from CIMMYT-Zimbabwe, have previously been reported to exhibit resistance to FAW in maize breeding studies. Among these, the first three lines were identified as resistant donor lines, whereas the fourth line, recognized as highly susceptible by CIMMYT, was included as a susceptible control [14].

### 2.3. Experimental Design

The resistance of maize genotypes to FAW was evaluated under both greenhouse and field conditions during the 2024/2025 growing season:

Greenhouse experiment: The greenhouse experiment was conducted under two environmental treatments: artificially infested and non-infested (control). A total of 20 maize genotypes were evaluated across benches serving as experimental units (Figure S1, Supplemental Materials), with plots consisting of three plants per genotype. Artificial infestation with fall armyworm larvae was performed to ensure uniform pest pressure in the infested treatment, while control plants were maintained without infestation. Although minor spatial differences among benches such as light intensity or airflow were possible, inspection of variance components indicated negligible heterogeneity among benches, confirming the relative uniformity of the greenhouse environment. Damage severity was assessed using the Davis 1–9 visual rating scale at multiple observation dates after infestation [1,8].

Field experiment: Field evaluation of maize genotypes was conducted using an alpha-lattice design (5 × 4) with two replications to improve the control of spatial variability within the experimental field. The experiment included two environments, infested and non-infested, to evaluate genotypic responses under different levels of pest pressure. Each replication was subdivided into incomplete blocks to reduce local field heterogeneity. Fall armyworm damage was assessed periodically using the Davis scale, and damage progression across observation dates was summarized using the area under the damage progress curve (*AUDPC*) [15].

### 2.4. Artificial Infestation and Damage Assessment

FAW larvae used for artificial infestation were collected from a maize field at Chókwè Seed-Co Company that had not received any insecticide treatment. The source field covered approximately 2 ha and was at the V6 growth stage (Figure S2, Supplemental Materials), when the experimental trial was at the V3 stage, figure besides, allowing the collection of larvae at different developmental stages ranging from eggs to adult moths.

Larvae were collected manually and placed individually in small bottles (Figure S3, Supplemental Materials) containing fresh maize leaves as food. The bottles were closed with cotton to allow ventilation and prevent escape. Each bottle contained a single larva to avoid cannibalism, which is common in fall armyworm populations. From the collected larvae, individuals at the second instar stage were selected based on similar body size and active movement to ensure uniform infestation across plants.

Artificial infestation was initiated when maize plants reached approximately the V4 growth stage. Infestation was performed three times at weekly intervals. During the first infestation, four larvae were placed into the whorl of each plant; during the second infestation, three larvae were applied; and during the third infestation, two larvae were introduced. Damage assessments were conducted six days after each infestation event. After the final infestation, two additional assessments were performed to monitor the progression of damage. All damage evaluations were conducted simultaneously in both the infested and control environments.

To maintain appropriate experimental conditions, insecticide application was planned for the non-infested treatments if natural fall armyworm infestation occurred. In the greenhouse experiment, insecticide application was not required because the control plants remained free of infestation despite their proximity to the infested treatment (Figure S4, Supplemental Materials). In contrast, insecticide application was necessary in the field control plots when the first signs of natural FAW infestation were observed, in order to maintain the intended non-infested condition.

Fall armyworm damage was assessed using the Davis 1–9 visual rating scale [8,16], where 1 represents no visible damage and 9 represents severe leaf damage or plant death (Figure S5, Supplemental Materials). Damage scores were recorded at multiple observation dates during crop growth to capture the progression of FAW injury. In this study, assessments were conducted at 25, 32, 39, 46, and 53 days after planting (DAP).

To summarize the progression of FAW damage over time, the *AUDPC* was calculated using the trapezoidal method described by [15]. *AUDPC* integrates both the intensity and duration of damage across observation dates and provides a quantitative measure of cumulative pest pressure. Lower *AUDPC* values indicate slower damage progression and therefore greater resistance to fall armyworm.

### 2.5. Data Preparation and AUDPC Calculation

Before statistical analysis, the dataset was examined to verify the structure of variables, detect missing values, and ensure that all variables were correctly formatted for analysis. FAW damage scores were recorded at multiple observation dates during crop growth using the Davis 1–9 visual rating scale, where 1 represents no visible leaf damage and 9 represents severe leaf damage or plant death [8,16].

To summarize the overall severity of FAW damage for each experimental unit, a mean damage score was calculated by averaging the recorded scores across all observation dates. The mean damage score was computed as:

$$\mathrm{Mean}\;\mathrm{Score}\;=\;\frac{\sum_{i=1}^{n}y_{i}}{n}$$

where $y_{i}$ represents the FAW damage score recorded at the $i^{th}$ observation date and n is the total number of observations.

To capture the progression of FAW damage over time, the *AUDPC* was calculated using the trapezoidal integration method described by [15]. The *AUDPC* provides a quantitative measure of cumulative pest damage across observation dates and integrates both the intensity and duration of damage. The *AUDPC* was calculated as:

$$AUDPC=\sum_{i=1}^{n-1}\frac{\left(y_{i}+y_{i+1}\right)}{2}\;(t_{i+1}-\;t_{i})$$

where $y_{i}$ = FAW damage score at the $i^{th}$ observation; $t_{i}$ = time (days after planting) at the $i^{th}$ observation; $(t_{i+1}-\;t_{i}$) = time interval between consecutive assessments; n = total number of observation dates. Lower *AUDPC* values indicate slower damage progression and therefore greater resistance to fall armyworm infestation.

According to the Davis damage rating scale, maize genotypes can be classified based on their mean FAW damage scores as resistant (scores 1–3), tolerant or moderately resistant (scores > 3–5), and susceptible (scores > 5–9) [1,8]. Although *AUDPC* values do not have fixed threshold ranges, lower *AUDPC* values indicate slower damage progression and therefore greater resistance to fall armyworm infestation, while higher *AUDPC* values reflect greater cumulative damage and increased susceptibility.

### 2.6. Statistical Analysis

All statistical analyses were conducted using R statistical software (R Core Team, 2024; version 4.3.2, R Foundation for Statistical Computing, Vienna, Austria) [17]. Prior to analysis, the dataset was examined to verify variable structure, identify missing values, and ensure that categorical variables such as genotype and environment were properly defined as factors. For the greenhouse experiment, fall armyworm damage progression, summarized as the area under the damage progress curve (*AUDPC*), was analyzed using a linear model with genotype as a fixed effect:

$$AUDPC=\;\mu +G_{i}+\varepsilon_{ij}$$

where μ represents the overall mean, $G_{i}$ is the effect of the *i*th genotype, and $\varepsilon_{ij}$ is the residual error term. Analysis of variance (ANOVA) was performed to test for significant differences among genotypes in their response to fall armyworm infestation.

For the field experiment, *AUDPC* values were analyzed using a mixed-effects model to account for environmental variation. The final model included genotype as a fixed effect and environment as a random intercept:

$$AUDPC=\;\mu +G_{i}+E_{j}+\varepsilon_{ij}$$

where $G_{i}$ represents the genotype effect and $E_{j}$ represents the random effect of environment. This approach allowed for the estimation of genotypic performance while accounting for environmental heterogeneity across experimental conditions.

Estimated marginal means for genotypes were calculated to compare resistance levels among entries. Genotypes were ranked based on their adjusted *AUDPC* values, where lower values indicate slower damage progression and therefore greater resistance to fall armyworm.

All models were fitted using the lme4 package, with significance tests for fixed effects obtained using lmerTest. Estimated marginal means were calculated using the emmeans package, and graphical visualization of genotype performance was performed using ggplot2.

The choice of statistical models was guided by the experimental design and the structure of variance components observed in each environment. In the greenhouse experiment, environmental conditions were highly controlled, and the inspection of variance components indicated negligible variability among experimental units (benches). This confirmed the uniformity of the experimental conditions and justified the use of a simple linear model with genotype as a fixed effect (*AUDPC* ~ Genotype), allowing for direct inference on genotypic differences.

In contrast, the field experiment was conducted under heterogeneous environmental conditions using an alpha-lattice design. Initial models including replication and block effects indicated negligible variance attributable to these factors, whereas environmental variation contributed substantially to differences in *AUDPC*. Consequently, a mixed-effects model was adopted, treating genotype as a fixed effect and environment as a random factor. This approach allowed for accurate estimation of genotypic performance while accounting for environmental heterogeneity and improving the reliability of statistical inference.

The use of different statistical models for greenhouse and field data therefore reflects the underlying experimental conditions and ensures that model assumptions are consistent with the structure of the data.

### 2.7. Molecular Marker Analysis

To complement phenotypic screening, molecular marker analysis was conducted using SNP data generated by SEQART Africa (Nairobi, Kenya). DNA extracted from seed samples representing each genotype was genotyped using Diversity Arrays Technology sequencing (DArTseq), a high-throughput genotyping platform that provides genome-wide SNP markers for genetic analysis [18].

Based on previous studies reporting genomic regions associated with FAW resistance in maize, SNP markers located on chromosomes 1, 3, 4, 5, 6, 8, and 9 were selected for analysis. These chromosomes have been reported to harbor QTL and genes associated with FAW resistance, reflecting the complex and quantitative nature of the trait [11,12].

All SNP markers located on these chromosomes were extracted from the full dataset, resulting in a subset of approximately 10,603 SNPs representing FAW-associated genomic regions. SNP allele data were matched to the evaluated genotypes for downstream analysis.

Allele states were coded as 0 (homozygous reference), 1 (heterozygous), and 2 (homozygous SNP allele). For analysis, the presence of SNP alleles (states 1 and 2) was considered as an indicator of allele variation, while 0 represented the reference allele. Missing values were excluded from the calculations.

For each genotype, the total number of SNP alleles and the proportion of SNP alleles across all analyzed markers were calculated. This proportion was used as a measure of genomic variation across FAW-associated regions.

To evaluate the relationship between SNP allele composition and phenotypic resistance, association analysis was performed using Pearson correlation, linear regression, and ANOVA, with the AUDPC as the response variable. This approach enables the assessment of marker–trait relationships and the validation of genomic regions associated with resistance traits [10].

## 3. Results

### 3.1. Fall Armyworm Damage Distribution

**Figure 2:**
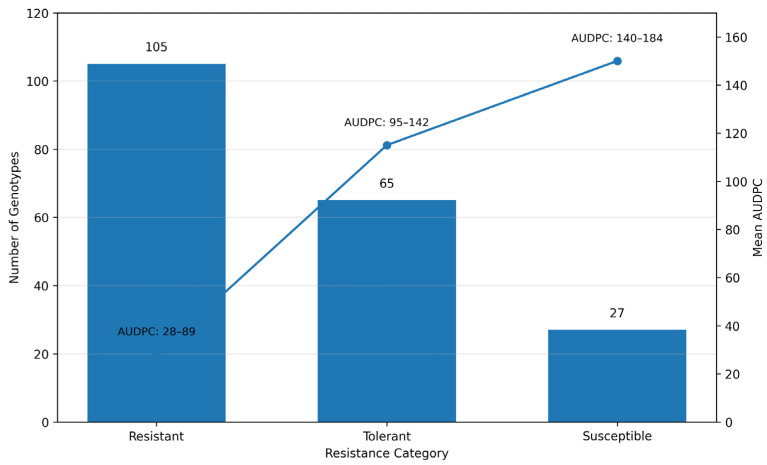
Distribution of maize genotypes classified according to FAW resistance categories based on the Davis scale, showing the number of genotypes and corresponding mean *AUDPC* values across resistance classes.

Exploratory analysis of FAW damage scores revealed clear differences in resistance levels among the evaluated maize genotypes. Based on the Davis damage rating scale, genotypes were classified into resistance categories using their mean damage scores. Among the evaluated observations, approximately 50.2% were classified as resistant (scores 1–3), 31.1% as tolerant (>3–5), and 12.9% as susceptible (>5–9), while a small proportion of records contained missing values (1.4%) (Figure 2).

The mean AUDPC values corresponded well with the Davis scale classification, indicating consistent trends between instantaneous damage scores and cumulative damage progression. Lower AUDPC values were associated with reduced FAW damage, indicating greater resistance among genotypes.

The distribution of genotypes across resistance classes is presented in Figure 2. A large proportion of genotypes were classified as resistant or tolerant, while a smaller proportion showed susceptibility to fall armyworm infestation. The corresponding *AUDPC* values followed a similar trend, confirming the consistency between mean damage scores and cumulative damage progression.

### 3.2. Differences Between Infested and Control Environments

**Figure 3:**
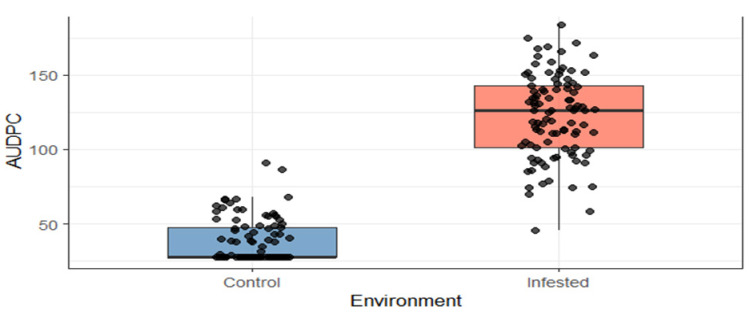
Distribution of *AUDPC* values across the control and infested environments, showing the contrast in FAW damage progression under protected and natural infestation conditions.

The distribution of *AUDPC* values differed substantially between control and artificially infested environments. Under control conditions, *AUDPC* values were generally low and clustered within a narrow range, reflecting minimal fall armyworm pressure. In contrast, the infested treatment showed substantially higher and more variable *AUDPC* values (Figure 3).

These results confirm that artificial infestation successfully increased damage intensity and improved discrimination among maize genotypes in terms of their resistance to FAW.

The distribution of *AUDPC* values under the control and infested conditions is shown in Figure 3. Under control conditions, *AUDPC* values were low and showed limited variability, whereas the infested treatment exhibited substantially higher and more variable *AUDPC* values. This indicates that artificial infestation effectively increased pest pressure and enhanced the differentiation of genotypic responses.

### 3.3. Greenhouse Genotypic Variation

Analysis of variance revealed significant differences among genotypes for FAW damage progression under controlled greenhouse conditions (F = 1.94, *p* = 0.012). This indicates the presence of substantial genetic variation in resistance to fall armyworm among the evaluated maize germplasm.

**Figure 4:**
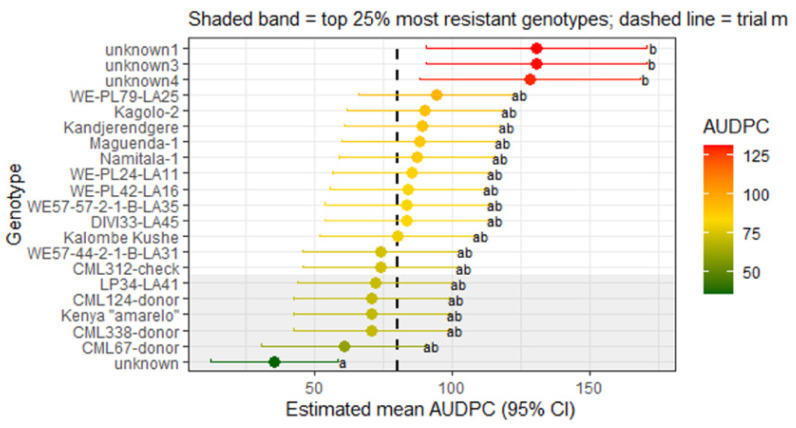
Estimated mean *AUDPC* values (±95% CI) for maize genotypes evaluated under greenhouse conditions. The shaded band represents the top 25% most resistant genotypes, and the dashed vertical line indicates the trial mean. Different letters (a, ab, b) indicate statistically significant differences among genotypes based on pairwise comparisons using estimated marginal means (*p* < 0.05).

Ranking of genotypes based on adjusted *AUDPC* values showed a continuous distribution of resistance levels rather than distinct resistant and susceptible groups (Figure 4). Several genotypes exhibited consistently lower *AUDPC* values, indicating slower damage accumulation and therefore stronger intrinsic resistance to FAW under controlled infestation conditions.

Figure 4 presents the ranked performance of maize genotypes under controlled greenhouse conditions based on adjusted *AUDPC* values. Genotypes with lower *AUDPC* values exhibited reduced damage progression and were therefore considered more resistant. The continuous distribution of values suggests that resistance is quantitatively inherited rather than controlled by a single major gene.

### 3.4. Field Genotypic Variation

Mixed-model analysis of field data accounting for environmental variation revealed significant genotypic differences in FAW resistance (*p* = 0.0217). Although environmental factors contributed to variation in *AUDPC* values, genetic differences among genotypes remained significant after accounting for these effects.

**Figure 5:**
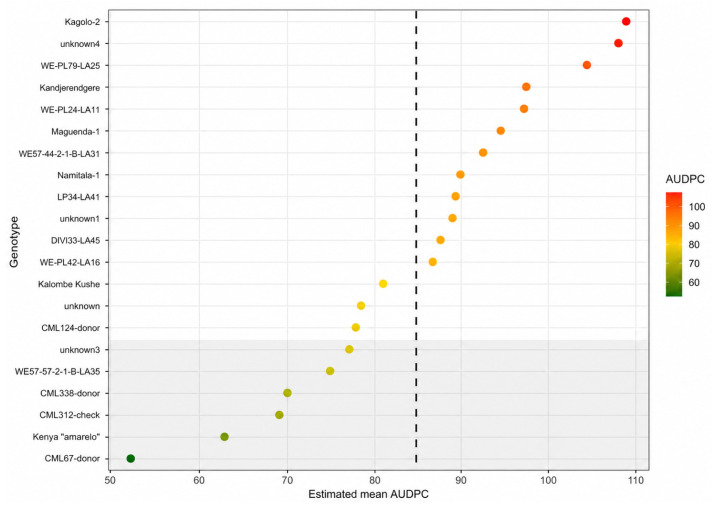
Estimated mean *AUDPC* values for maize genotypes evaluated under field conditions. The shaded band represents the most resistant genotypes (*AUDPC* ≤ 25th percentile, 76.9), and the dashed vertical line indicates the trial mean.

Compared with the greenhouse experiment, the range of *AUDPC* values in the field was narrower, reflecting the influence of environmental variability on resistance expression. Nevertheless, some genotypes consistently exhibited lower *AUDPC* values than the trial mean, indicating improved resistance under natural conditions (Figure 5).

The adjusted genotype means for *AUDPC* under field conditions are presented in (Figure 5). Compared with the greenhouse, the variation among genotypes was less pronounced, reflecting the influence of environmental factors on resistance expression. However, several genotypes maintained lower *AUDPC* values, indicating consistent performance under natural conditions.

### 3.5. Stability Across Environments

**Figure 6:**
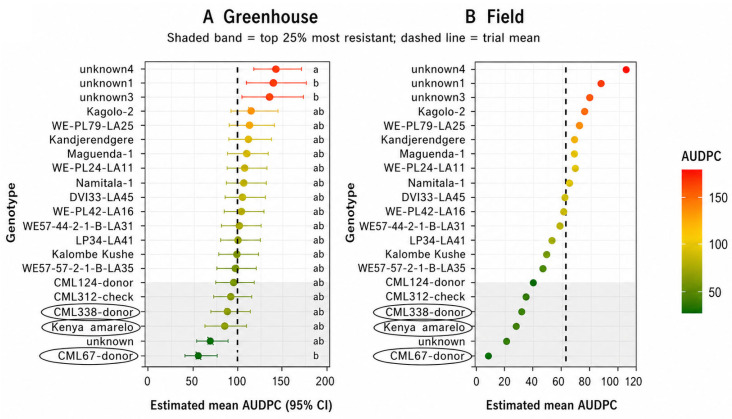
Comparative ranking of maize genotypes based on estimated mean *AUDPC* values under (**A**) greenhouse and (**B**) field conditions. The shaded band indicates the top 25% most resistant genotypes, and the dashed vertical line represents the trial mean in each environment. Different letters (a, ab, b) indicate statistically significant differences among genotypes based on pairwise comparisons using estimated marginal means (*p* < 0.05).

Comparison of genotype performance across greenhouse and field experiments identified a subset of genotypes that consistently exhibited low *AUDPC* values in both environments. These genotypes demonstrated stable resistance to fall armyworm infestation and therefore represent promising candidates for use as parental material in maize breeding programs targeting FAW resistance (Figure 6). The observed distribution of resistance across genotypes suggests that FAW resistance in the evaluated germplasm is quantitatively inherited and influenced by environmental conditions.

The circled genotypes indicate entries with consistently low *AUDPC* values across both environments, representing stable resistance, as we can see in Figure S6 (Supplemental Materials).

### 3.6. Molecular Marker Results

#### 3.6.1. SNP Distribution

The relationship between phenotypic resistance and SNP allele composition across FAW-associated chromosomes was evaluated and is presented in Table 2. Genotypes showed variation in SNP allele proportion, reflecting differences in genomic composition across candidate resistance regions.

Table 2 shows the relationship between fall armyworm damage (*AUDPC*), genotype ranking, and SNP allele proportion across chromosomes associated with FAW resistance. SNP allele proportion represents the fraction of non-reference alleles across all analyzed markers.

However, no clear correspondence was observed between SNP allele proportion and resistance ranking. For example, the most resistant genotype (CML67) exhibited a low SNP allele proportion, whereas several genotypes with higher allele proportions did not show improved resistance. This lack of agreement between molecular and phenotypic data suggests that general SNP variation across FAW-associated chromosomes does not directly explain resistance.

#### 3.6.2. Association Analysis

A total of 10,603 SNP markers located on chromosomes previously associated with FAW resistance were analyzed to evaluate the relationship between genomic allele composition and phenotypic resistance. The proportion of SNP alleles varied among genotypes, reflecting differences in genomic composition across FAW-associated regions.

**Figure 7:**
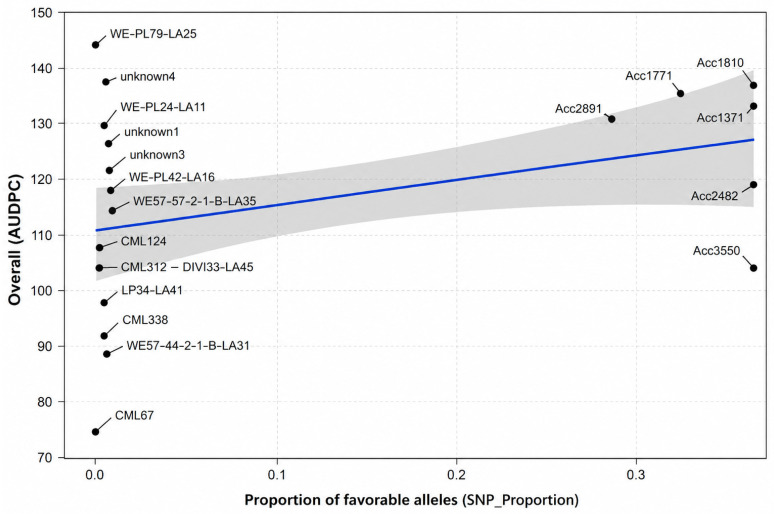
Association between allele proportion and FAW resistance.

Pearson correlation analysis revealed a positive but non-significant relationship between the proportion of SNP alleles and FAW damage (r = 0.34, *p* = 0.147), indicating that allele composition did not significantly influence resistance levels (Figure 7).

Similarly, linear regression analysis showed that the proportion of SNP alleles was not a significant predictor of *AUDPC* (β = 40.05, *p* = 0.147), explaining only 11.3% of the observed variation among genotypes (R^2^ = 0.113).

**Figure 8:**
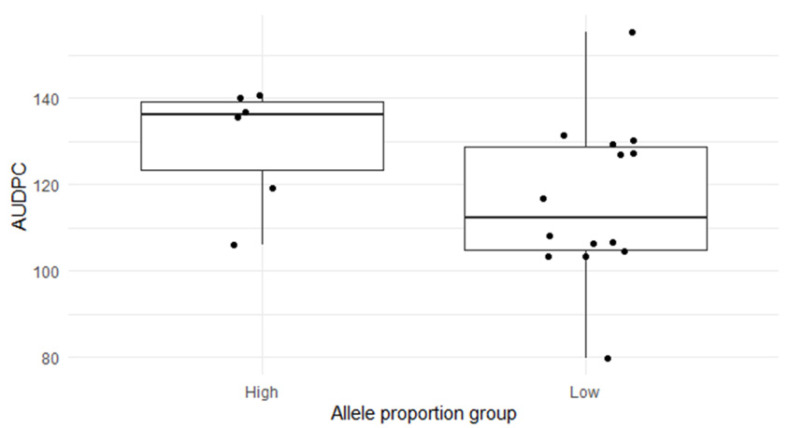
Comparison of *AUDPC* values between maize genotypes grouped by high and low SNP allele proportion.

Analysis of variance comparing genotypes with high and low allele proportions (Figure 8) also showed no significant differences in FAW damage (*p* = 0.133), further confirming the lack of association between SNP allele composition and phenotypic resistance.

These results indicate that genome-wide SNP variation across FAW-associated chromosomes is not sufficient to explain resistance in the evaluated maize germplasm. In addition, FAW resistance is not determined by overall allele composition across candidate genomic regions, but is likely controlled by specific loci or complex gene interactions not captured by this analysis.

Figure 7 shows the relationship between SNP allele proportion across FAW-associated chromosomes and *AUDPC* in maize genotypes. The regression analysis showed a positive but non-significant relationship (r = 0.34, *p* = 0.147), indicating that allele composition does not significantly explain variation in resistance.

Figure 8 shows a comparison of *AUDPC* between maize genotypes grouped by SNP allele proportion. Genotypes were classified into high and low allele proportion groups based on SNP variation across chromosomes associated with FAW resistance. Boxplots show the median and interquartile range, with individual data points representing genotypes. Despite observable variation within groups, no significant differences were detected (*p* > 0.05), suggesting that genomic SNP allele composition does not explain phenotypic resistance.

## 4. Discussion

The present study demonstrated clear variation in FAW resistance among maize genotypes evaluated under both greenhouse and field conditions. The continuous distribution of *AUDPC* values across genotypes indicates that resistance is quantitatively inherited rather than controlled by a single major gene. Importantly, in the context of insect resistance, no genotype can be considered completely immune to FAW infestation. Rather, the observed differences among genotypes reflect varying levels of resistance, whereby some genotypes possess a greater natural ability to tolerate or limit pest damage than others. This concept is consistent with the traditional host plant resistance (HPR) framework [19], which describes three major resistance mechanisms: antixenosis (non-preference), antibiosis, and tolerance. In maize, these mechanisms may involve pest avoidance, active plant defense responses, or the ability to maintain growth and yield despite infestation. More recent studies have further expanded HPR concepts to include biochemical defenses such as secondary metabolites, genetic mechanisms including quantitative trait loci (QTL), and tritrophic interactions involving plants, pests, and their natural enemies [20,21].

This pattern is consistent with previous studies reporting that FAW resistance in maize is governed by multiple loci with small additive effects and QTL distributed across several chromosomes [1,8,9,11,12]. Such quantitative resistance is typically associated with a combination of morphological and physiological mechanisms, including leaf toughness, antibiosis, and tolerance responses that reduce the impact of insect feeding. Similar quantitative inheritance patterns for insect resistance in maize have also been reported by [22,23], reinforcing the complex genetic architecture of the trait.

Differences observed between greenhouse and field evaluations highlight the important role of environmental conditions in the expression of FAW resistance. Under greenhouse conditions, where infestation pressure was uniform and environmental variability minimized, genotypic differences were more clearly expressed, resulting in a wider range of *AUDPC* values. In contrast, field conditions introduced additional sources of variation, including spatial heterogeneity, fluctuating pest pressure, and genotype × environment interactions, which reduced the magnitude of observable differences among genotypes. Similar observations have been reported by [8,9,22,23], emphasizing that controlled greenhouse screening improves the detection of intrinsic resistance, whereas field evaluation captures genotype × environment interactions under natural infestation.

Despite environmental influences, several genotypes exhibited consistent performance across both environments, indicating stable resistance to FAW. Genotypes such as CML67, CML338, and Kenya amarelo (Acc3550) maintained relatively low *AUDPC* values under both greenhouse and field conditions. The stability of these genotypes suggests the presence of robust resistance mechanisms that are less sensitive to environmental variation. From a breeding perspective, such stability is highly desirable, as it ensures reliable performance across diverse production environments. The identification of these stable resistant genotypes has important implications for maize improvement programs at IIAM, as they represent valuable parental material for the development of improved varieties combining FAW resistance with local adaptation. Furthermore, the integration of CIMMYT-derived resistant lines with IIAM germplasm provides an opportunity to broaden the genetic base of resistance while maintaining adaptation to Mozambican agroecological conditions.

In contrast to the clear phenotypic differentiation among genotypes, molecular marker analysis did not reveal a significant association between SNP allele composition and FAW resistance. Although a large number of SNP markers located on chromosomes previously associated with FAW resistance were analyzed, the proportion of SNP alleles did not significantly explain the variation in *AUDPC*. This lack of association suggests that general genomic variation across candidate regions is not sufficient to predict resistance. Similar limitations of broad marker-based prediction for insect resistance traits have been reported by [1,11,12], who highlighted that resistance is often explained by specific QTL rather than genome-wide allele proportions.

The moderate but non-significant correlation observed between SNP allele proportion and FAW damage likely reflects background genomic variation rather than functional genetic effects. Several factors may explain this result. First, recombination between markers and causal genes may reduce linkage, limiting the predictive power of the selected markers. Second, differences in genetic background between populations may affect the expression of resistance loci. Third, FAW resistance is likely controlled by complex gene networks and interactions that are not captured by simple allele proportion metrics. These findings reinforce the concept that FAW resistance in maize is a complex polygenic trait. Even with the analysis of thousands of SNP markers across relevant chromosomes, the absence of significant associations highlights the limitations of using general genomic variation as a proxy for resistance.

For maize breeding programs in Mozambique, these findings suggest that phenotypic selection remains essential, particularly under controlled infestation conditions where genotypic differences can be reliably detected. Molecular tools should therefore be used as complementary approaches rather than replacements for phenotypic screening. Future studies should adopt more targeted genomic approaches to improve the detection of loci associated with FAW resistance in maize. In particular, the use of extreme phenotype groups contrasting highly resistant and highly susceptible genotypes may enhance the power to detect significant SNP associations and facilitate the identification of candidate loci for marker-assisted selection. In addition, expanding SNP screening to include additional chromosomes and broader genome-wide marker coverage will be important for capturing loci that may contribute to resistance beyond the candidate regions assessed in the present study. Such approaches are expected to provide deeper insight into the complex polygenic architecture of FAW resistance and strengthen the application of molecular tools in maize breeding programs.

In the context of Mozambique, where maize production is frequently affected by multiple stresses, including drought and FAW infestation, the development of resilient varieties is particularly important. This observation is highly relevant across Sub-Saharan Africa, where FAW and drought frequently co-occur and jointly reduce maize productivity [1,3,4]. Therefore, breeding strategies should aim to combine FAW resistance with tolerance to abiotic stresses to ensure stable productivity, resilience, and food security in smallholder farming systems.

Overall, this study confirms that FAW resistance in maize is governed by complex genetic and environmental interactions. The combined use of controlled screening, field evaluation, and molecular analysis provides a comprehensive framework for identifying resistant germplasm. The genotypes exhibiting stable resistance across environments represent promising candidates for use in breeding programs aimed at improving FAW resistance and enhancing food security in Mozambique and the broader Sub-Saharan African region.

## 5. Conclusions

This study demonstrated the presence of significant genetic variation for fall armyworm resistance among maize genotypes under controlled greenhouse conditions, confirming the effectiveness of phenotypic screening in identifying resistant germplasm. Although environmental effects influenced resistance expression in the field, several genotypes exhibited stable performance across environments, highlighting their potential as parental material for breeding programs.

In contrast, genome-wide SNP variation across chromosomes previously associated with FAW resistance did not explain phenotypic differences among genotypes. This finding indicates that FAW resistance is a complex, quantitatively inherited trait controlled by specific loci and gene interactions rather than overall allele composition.

These results emphasize the continued importance of phenotypic selection, supported by targeted genomic approaches, for the development of FAW-resistant maize varieties adapted to Mozambique and similar agroecological systems.

## Figures and Tables

**Table 1:** Maize genotypes evaluated in the study including IIAM and CIMMYT germplasm.

No.	Genotype (Field Name)	Genotype (Molecular Analysis Name)	Type	Source	Program/Origin	Remarks
1	WE57-44-2-1-B-LA31	WE57-44-2-1	Inbred line	IIAM	WEMA	Local breeding line
2	LP34-LA41	LP34	Inbred line	IIAM	INIA	Local breeding line
3	Kenya amarelo (Acc3550)	Acc3550	Accession	IIAM	Genebank	Stable resistant genotype
4	DIVI33-LA45	DIVI33	Inbred line	IIAM	PASS	Local breeding line
5	WE57-57-2-1-B-LA35	WE57-57-2-1	Inbred line	IIAM	WEMA	Local breeding line
6	Kalombe Kushe (Acc2482)	Acc2482	Accession	IIAM	Genebank	Local accession
7	unknown3	unknown3	Landrace	IIAM	Genebank	Traditional germplasm
8	WE-PL42-LA16	WE-PL42	Inbred line	IIAM	WEMA	Local breeding line
9	unknown1	unknown1	Landrace	IIAM	Genebank	Traditional germplasm
10	unknown4	unknown4	Landrace	IIAM	Genebank	Traditional germplasm
11	WE-PL24-LA11	WE-PL24	Inbred line	IIAM	WEMA	Local breeding line
12	Kagolo-2 (Acc2891)	Acc2891	Accession	IIAM	Genebank	Local accession
13	Namitala-1 (Acc1371)	Acc1371	Accession	IIAM	Genebank	Local accession
14	Kandjerendgere (Acc1810)	Acc1810	Accession	IIAM	Genebank	Local accession
15	Maguenda-1 (Acc1771)	Acc1771	Accession	IIAM	Genebank	Local accession
16	WE-PL79-LA25	WE-PL79	Inbred line	IIAM	WEMA	Local breeding line
17	CML67	CML67	Inbred line	CIMMYT	Donor line	Stable resistant genotype
18	CML338	CML338	Inbred line	CIMMYT	Donor line	Stable resistant genotype
19	CML124	CML124	Inbred line	CIMMYT	Donor line	Resistant donor
20	CML312	CML312	Check line	CIMMYT	Susceptible control	Susceptible check

**Table 2:** Relationship between phenotypic resistance and SNP allele composition across FAW-associated chromosomes.

Genotype	Field	Screen	Overall	Rank	Favorable alleles	Total markers	Proportion favorable	Marker group
CML67	66.5	92.94444444	79.72222222	1	0	0	0	Low
CML338	92.75	114.1388889	103.4444444	2	0	0	0	Low
WE57-44-2-1-B-LA31	110.8333333	96.25	103.5416667	3	0	0	0	Low
LP34-LA41	115.9375	93.13888889	104.5381944	4	0	0	0	Low
ACC3550	88.2	123.6666667	105.9333333	5	55	168	0.327380952	High
CML312	85.75	127.1666667	106.4583333	6	0	0	0	Low
DIVI33-LA45	120.1375	92.94444444	106.5409722	7	0	0	0	Low
CML124	111.125	105	108.0625	8	0	0	0	Low
WE57-57-2-1-B-LA35	95.8125	137.6666667	116.7395833	9	0	0	0	Low
ACC2482	113.6041667	124.8333333	119.21875	10	49	144	0.340277778	High
UNKNOWN3	108.7625	145.0555556	126.9090278	11	0	0	0	Low
WE-PL42-LA16	124.775	130.0833333	127.4291667	12	0	0	0	Low
UNKNOWN1	120.8375	137.6666667	129.2520833	13	0	0	0	Low
UNKNOWN4	139.65	120.9444444	130.2972222	14	0	0	0	Low
WE-PL24-LA11	136.5	126.5833333	131.5416667	15	0	0	0	Low
ACC2891	142.9166667	128.5277778	135.7222222	16	41	154	0.266233766	High
ACC1371	130.6083333	143.1111111	136.8597222	17	52	153	0.339869281	High
ACC1810	140.35	140	140.175	18	61	179	0.340782123	High
ACC1771	140.7875	140.3888889	140.5881944	19	45	148	0.304054054	High
WE-PL79-LA25	163.0416667	147.7777778	155.4097222	20	0	0	0	Low

## Data Availability

The data presented in this study are available from the corresponding author upon reasonable request. The data are not publicly available due to ongoing analyses and institutional data-sharing policies.

## Supplementary Materials

The following supporting information can be downloaded at: https://www.mdpi.com/article/10.3390/genes17050526/s1, Figure S1: Screenhouse bench distribution; Figure S2: FAW collection and infestation process; Figure S3: Larvae bottle handling and size selection; Figure S4: Screenhouse control and infested plants; Figure S5: Representative FAW damage; Figure S6: Comparative ranking of maize genotypes based on estimated mean AUDPC values under greenhouse and field conditions, highlighting genotypes with stable resistance across environments. The stable genotypes name: CML67, Kenya amarelo (Acc3550), CML338, respectively.
