# Supplementary material for: Screening Maize Germplasm for Resistance to Fall Armyworm (Spodoptera frugiperda) and Its Association with Genomic SNP Variation
Source: Genes (Basel). 2026 Apr 29;17(5):526. doi: 10.3390/genes17050526 (PMC13205618; doi:10.3390/genes17050526)
Supplement: Supplementary file 1 [file genes-17-00526-s001.zip › genes-4247874-supplementary.pdf]

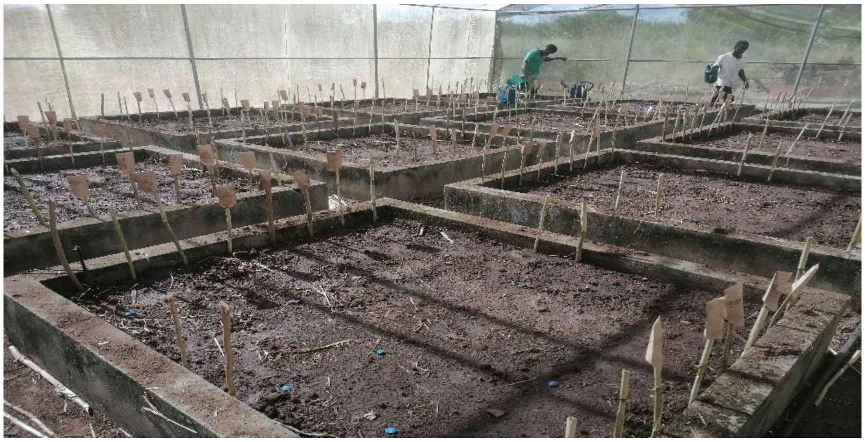

Figure S1: Screenhouse bench distribution

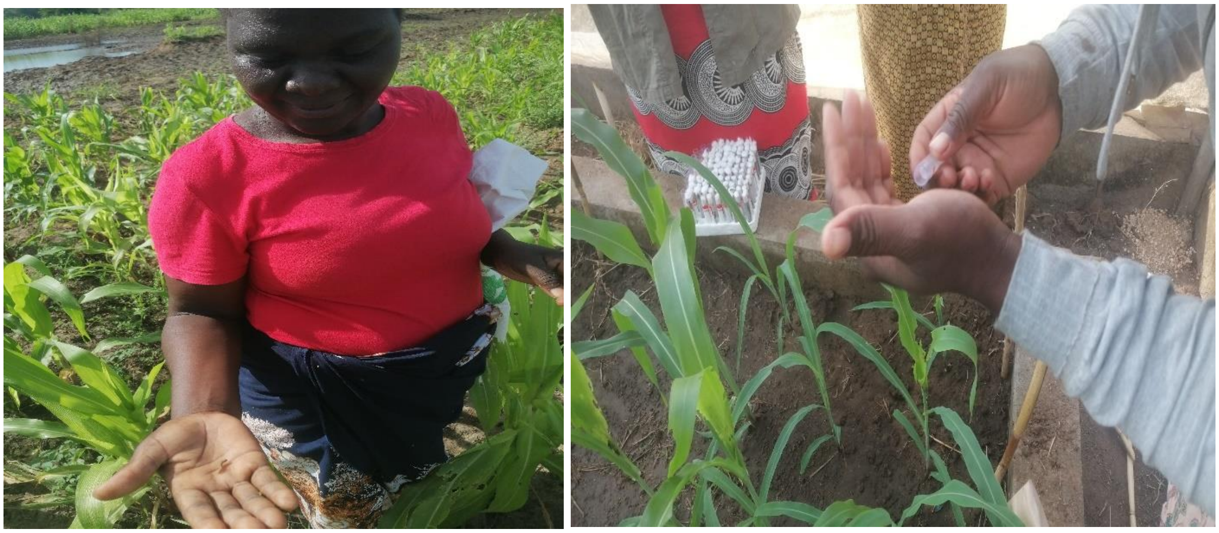

Figure S2: FAW collection and infestation process

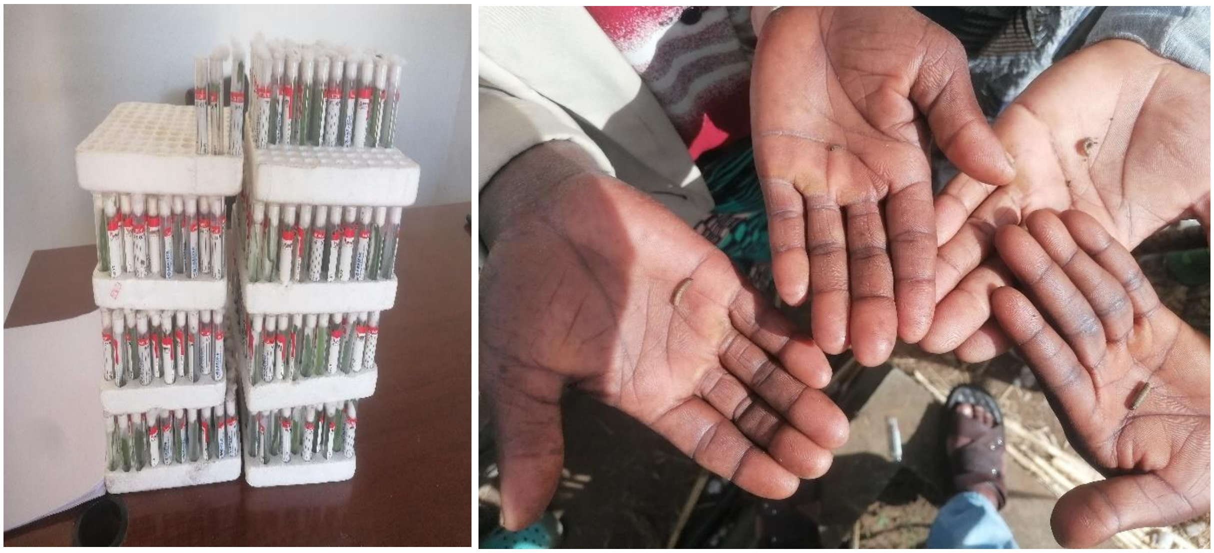

Figure S3: Larvae bottle handling and size selection

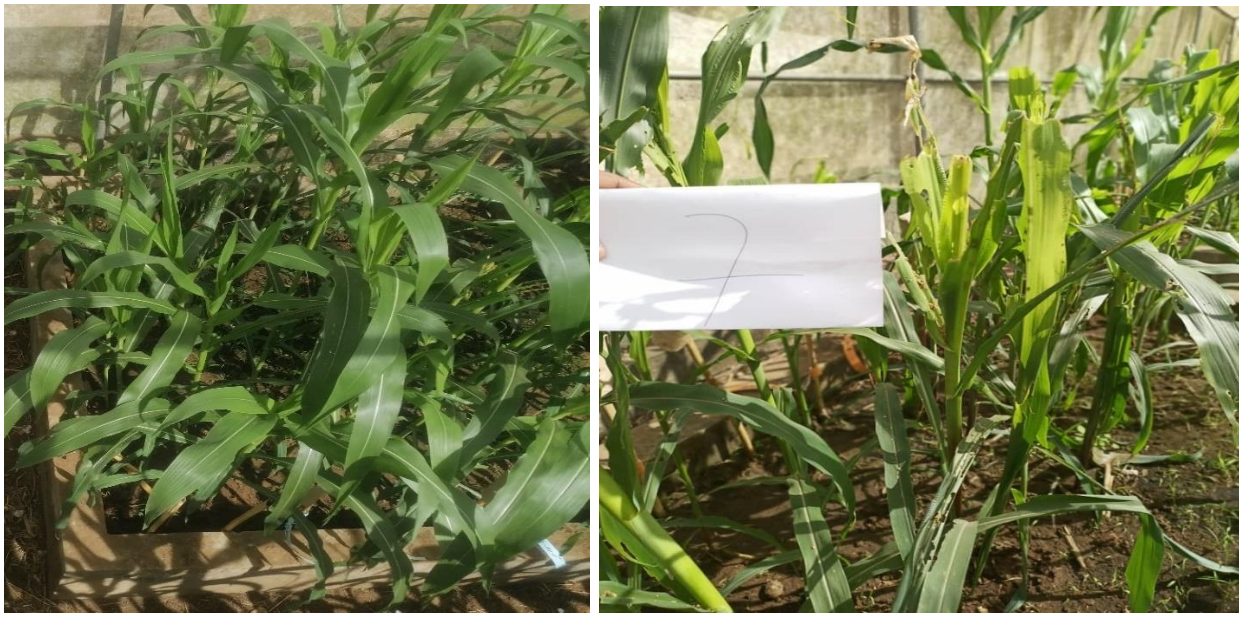

Figure S4: *Screenhouse control and infested plants*

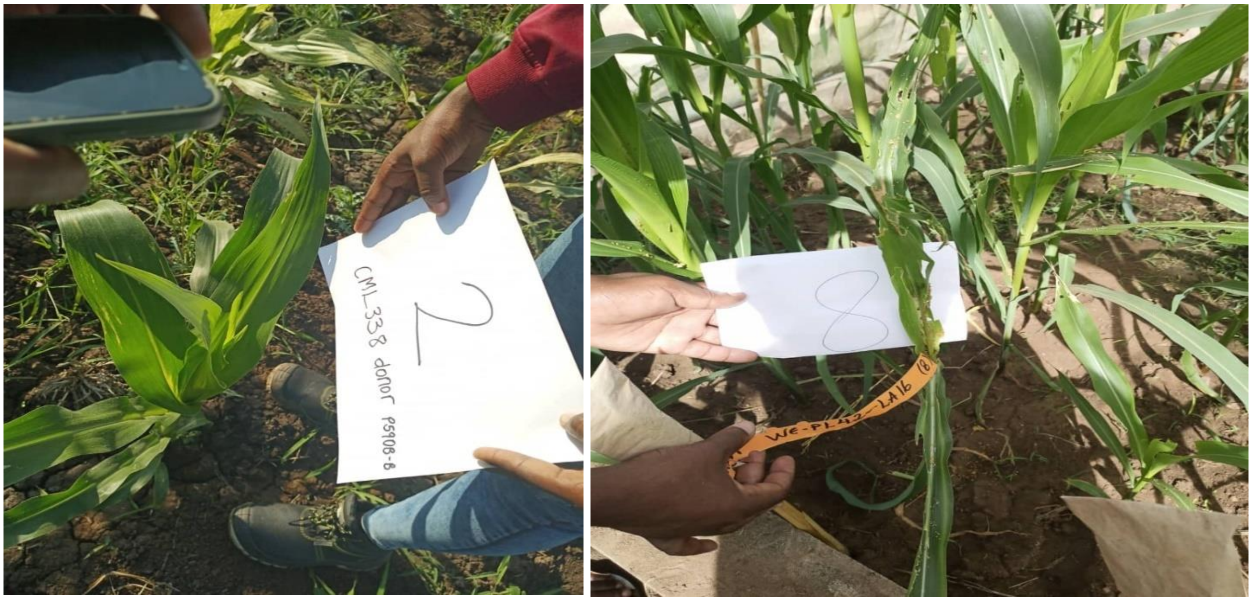

Figure S5: *Representative FAW damage*

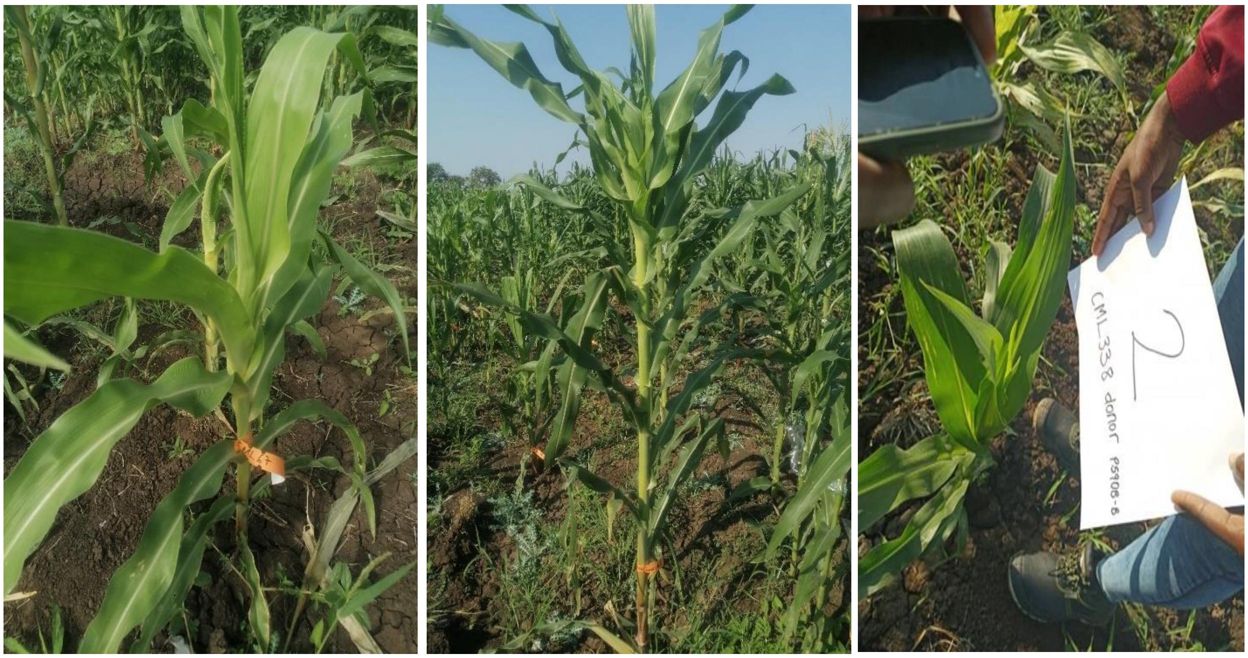

Figure S6: Comparative ranking of maize genotypes based on estimated mean AUDPC values under greenhouse and field conditions, highlighting genotypes with stable resistance across environments. The stable genotypes name: CML67, Kenya amarelo (Acc3550), CML338, respectively
